# Beyond research: a primer for considerations on using viral metagenomics in the field and clinic

**DOI:** 10.3389/fmicb.2015.00224

**Published:** 2015-03-25

**Authors:** Richard J. Hall, Jenny L. Draper, Fiona G. G. Nielsen, Bas E. Dutilh

**Affiliations:** ^1^Institute of Environmental Science and Research, National Centre for Biosecurity and Infectious Disease, Upper HuttNew Zealand; ^2^Ministry for Primary Industries Animal Health Laboratory, National Centre for Biosecurity and Infectious Disease, Upper HuttNew Zealand; ^3^DNAdigest, Future Business Centre, CambridgeUK; ^4^Theoretical Biology and Bioinformatics, Utrecht University, UtrechtNetherlands; ^5^ Centre for Molecular and Biomolecular Informatics, Radboud Institute for Molecular Life Sciences, Radboud University Medical Centre, NijmegenNetherlands; ^6^Department of Marine Biology, Institute of Biology, Federal University of Rio de Janeiro, Rio de JaneiroBrazil

**Keywords:** viral metagenomics, ethics, medical, metagenomics, data interpretation, incidental findings, diagnostic tools, biomarkers

## Abstract

Powered by recent advances in next-generation sequencing technologies, metagenomics has already unveiled vast microbial biodiversity in a range of environments, and is increasingly being applied in clinics for difficult-to-diagnose cases. It can be tempting to suggest that metagenomics could be used as a “universal test” for all pathogens without the need to conduct lengthy serial testing using specific assays. While this is an exciting prospect, there are issues that need to be addressed before metagenomic methods can be applied with rigor as a diagnostic tool, including the potential for incidental findings, unforeseen consequences for trade and regulatory authorities, privacy and cultural issues, data sharing, and appropriate reporting of results to end-users. These issues will require consideration and discussion across a range of disciplines, with inclusion of scientists, ethicists, clinicians, diagnosticians, health practitioners, and ultimately the public. Here, we provide a primer for consideration on some of these issues.

## Introduction

Efforts to discover and describe new viral species have lagged behind other organisms such as bacteria, fungi, or eukaryotes (Rosario and Breitbart, 2011; Delwart, 2013; Hurwitz and Sullivan, 2013). In part, this is due to the lack of a universal conserved genetic element shared between viral genomes which could be exploited for the purposes of viral genome discovery. For other microbes, there are conserved elements which can be targeted to detect new taxa, such as the 16S ribosomal RNA (rRNA) gene for bacteria, or the internal transcribed spacer (ITS) region for fungi. Next-generation sequencing in shotgun metagenomics has greatly increased the capacity for, and discovery of, new viruses (Mokili et al., 2012). This topic has been the subject of intense scientific effort and review since the first application of this technology in 2002 to describe uncultured viruses in the marine environment (Breitbart et al., 2002). Given the common application of metagenomics for virus discovery in basic and applied research, it is not surprising that there is a growing interest for use in a diagnostic capacity. Potential diagnostic applications of viral metagenomics span many areas, from horticulture (Kehoe et al., 2014) to veterinary medicine (Blomstrom, 2011; Belak et al., 2013) through to human health (Miller et al., 2013). Similarly, metagenomics can be used as a tool in forensics (Karlsson et al., 2013) and for monitoring environmental samples such as water quality (Ng et al., 2012).

### A Universal Test: Incidental Findings

In the example of human health, diagnostic tests traditionally proceed as follows. First, a clinician examines a patient and makes a provisional diagnosis, which may then require further laboratory testing. If an infectious disease is suspected, the clinician is likely to request that pathogen A, B, or C be tested for, and a diagnostic laboratory will use culture, antigen, or antibody detection (immunoassay), or molecular methods. This process is similar for the diagnosis of infectious disease in animals or plants. Syndromic diseases, such as influenza-like respiratory illness in humans, vesicular disease in livestock, or “virus-like symptoms” in plants may require a more complex diagnostic workup, which could take place as a multiplex PCR or parallel/serial specific testing. In general terms, most PCR, culture, or immunoassay-based diagnostic methods only provide evidence for the presence or absence of specific pathogens. This is often all that is required to enable appropriate therapy. Additional techniques may be needed if other attributes of a pathogen are to be determined, i.e., antiviral or antimicrobial resistance, or presence of virulence genes/toxins. In contrast, metagenomics has the potential to detect all known organisms within a sample in a single experiment, and is particularly useful for viruses given the lack of a universally conserved genetic element. Deep metagenomic sequencing has the potential to not only identify viruses with a high level of taxonomic resolution, but may also reveal other attributes that are clinically relevant, such as resistance to antiviral medications (Quinones-Mateu et al., 2014). Because of these advantages, metagenomics is being increasingly applied in diagnostics, and is already being offered as a commercial diagnostic service^1^^,^^2^ .

Thus far, the potential ethical implications of metagenomics to detect all organisms in a sample have received less attention, with a notable absence of discussion in relation to the detection and diagnosis of pathogens. For example, the technique has an inherent capability for incidental detection of pathogens that may not be relevant to the condition for which the patient is seeking treatment. It is therefore prudent to consider the possible impacts of an incidental diagnosis before proceeding with such a test. Consider the hypothetical example of a stool sample sent to a medical laboratory for norovirus testing. If the patient is HIV-positive and metagenomics is applied, then it is likely that HIV sequences will be detected in the metagenomic data (especially relevant for the RNA metagenome, also known as the metatranscriptome), given that up to 60% of HIV patients have detectable levels of HIV in their stool (van der Hoek et al., 1995). Both patient and physician need to be aware of this potential. In cases where such incidental findings are unwanted, a technical solution is possible: using bioinformatics, a panel of high-consequence viruses (or pathogens of concern) could be rapidly filtered from the data before reporting took place.

The potential for incidental findings may seem alarming, but is not unprecedented. Technologies such as magnetic resonance imaging (MRI) or computer-tomography (CT) scanning can detect cancer as an incidental finding, but patients will usually be made aware of this possibility before consenting to a test. The physician will have a clear understanding of how to manage the finding of malignancy, should it be observed. Moreover, we observe a parallel with individualized complete human genome or exome sequencing, where an average human being carries approximately 250–300 loss-of-function variants in annotated genes, and 50–100 variants previously implicated in inherited disorders (Genomes Project et al., 2010). We contend that it is the responsibility of any researcher or diagnostician who uses metagenomics in a clinical setting to ensure that both the physician and patient are aware of the potential for detection of any pathogen, even those unrelated to their disease, and that informed consent information is clear about the processes in place for supporting the patient in case of incidental findings. There are, however, to our knowledge, not yet any established guidelines for how such informed consent should be handled in the case of clinical diagnosis using metagenomic assays, which makes the requirement of informed consent very difficult to implement in practice for individual testing labs.

For clinical exome or whole-genome sequencing of patients, recommendations on incidental findings have been developed by the American College of Medical Genetics and Genomics (ACMG), including: a “minimum list” of serious disease variants which are recommended to be reported from the genetic lab to the ordering clinician; a recommendation on the type of (likely) pathogenic incidental findings to report; guidelines for pre- and post-test counseling of the patient; and recommendations for a patient to opt-out of receiving incidental findings (Green et al., 2013). Guidelines for consent, processing and reporting of incidental findings for clinical metagenomic sequencing addressing the above issues are urgently required to ensure that adequate ethical considerations are met when handling and analyzing human metagenomic data.

## The Host Genome: Culture, Ethnicity, and the Law

A metagenomic dataset is likely to contain significant amounts of host genome sequence, depending on the sample type and sequencing protocol. Routine clinical samples in human health such as feces, swabs, tissue biopsy, sputum, and urine, are all likely to contain human DNA and RNA. In some countries, there are strict legal controls in place that govern the use of human tissue. For example, in New Zealand, consent of the patient for the use of their tissue for any future unspecified research purposes is an absolute statutory requirement (Ministry of Health, 2007), and the tissue may only be used for the express intention for which it was collected (New Zealand Government, 2008). In particular, the indigenous people of New Zealand, Māori, have a unique cultural perspective on the ownership of human genetic information, which is not held to be the property of the individual, and decisions about investigating DNA should be made collectively (Baird et al., 1995; Hudson, 2009; New Zealand Health Research Council, 2010). Thus, it is possible that the incidental sequencing of human DNA could lead to legal, ethical, and/or cultural obligations when conducting a metagenomic analysis. Of course, the controls around sequencing of human DNA will vary greatly between jurisdictions, but the implications of generating incidental information should be accounted for. Once again, technical solutions such as bioinformatic filtering of the data for human DNA may provide a solution, but there would need to be some confidence around the quality of this process for deleting human genomic sequences. Without the removal of human sequence, human metagenomic datasets are likely to be subject to legislation for health-related personal data, which implies requirements for subject anonymization and restricted data access (Mascalzoni et al., 2014). To ensure the highest level of sharing and deposition of metagenomic data in research data repositories, we urge repositories and ethical review committees to develop guidelines on which methods are considered adequate for the removal of potentially personally identifiable human sequences from metagenomic data sets, to allow for deposition and sharing of metagenomic data without restrictions. Comprehensive guidelines should take into consideration both the benefit of data usage to inform research and any potential harm for patients as a result of non-compliance to guidelines. Consequences for a breach of ethical approval, and procedures to recall shared data in the event of a breach should also be considered. The ethical considerations for developing governance and guidelines have been discussed in depth by the Nuffield Council on Bioethics in their report concerning the collection and usage of biomedical data for research and health care (Nuffield Council on Bioethics, 2014).

## Trade Implications

The detection of pathogens of agricultural or horticultural significance can have dire consequences for productivity and trade. Most nations have government veterinary or plant health laboratories that provide testing and surveillance for significant pathogens. These laboratories specialize in screening animals and goods for trade purposes, preventing the spread of pathogens, and playing a crucial role in demonstrating that a country or region is free of specific diseases. In the case of trade in animals and animal products, the World Organization for Animal Health (OIE) maintains a list of the veterinary diseases of greatest concern worldwide, tracks, and reports on their global occurrence, and sets diagnostic testing standards for these diseases to aid international trade. New outbreaks of OIE-listed diseases in countries previously considered free of the disease can have severe trade implications, resulting in either blockage of relevant trade permits, or a massive increase in the testing and documentation required to certify products for export. The cost of such outbreaks can greatly affect trade-based economies, and proving freedom from such a disease once it is detected (or even suspected) can be a major economic burden.

Due to the sensitivity and untargeted nature of metagenomics, its application to animal or plant samples presents a challenge for regulatory authorities (MacDiarmid et al., 2013). Methods for assigning metagenomic sequencing reads to species may not be perfect, and incomplete reference databases as well as evolutionary conservation between species can easily lead to an incorrect diagnosis. Additionally, genetic databases are still skewed towards heavily researched organisms – particularly pathogens – and this bias increases the chance of relatively benign viruses being classified as “similar to” viruses of significant agricultural or clinical concern. A hypothetical example of a fish metagenome can be used to illustrate this point. Such a metagenome could conceivably contain sequences from a relatively benign virus in the family *Orthomyxoviridae*, which has some genetic similarity to the trade-sensitive *Isavirus* that causes Infectious Salmon Anemia (ISA) in salmon. If such a sequence, found in a healthy fish, is reported as “similar to ISA virus,” this finding could have significant implications for trade in salmon, given that even the suggestion that an OIE-listed virus may be present can be enough to impose significant trade restrictions on an exporting country.

Responsibility must be taken by researchers and diagnosticians performing metagenomic analyses to ensure the veracity of their results and to engage with regulatory authorities early on in the process once an initial discovery has been made, to avoid unintended economic harm from falsely declaring the presence of an organism with trade implications.

## Use of the Data

It is important that metagenomic datasets are freely and openly shared upon the publication of scientific results, for example by deposition in a database such as EBI Metagenomics (Hunter et al., 2014), MG-RAST (Meyer et al., 2008), or Genbank (Benson et al., 2014). This allows other researchers to investigate the data, to substantiate or refute claims made by the original authors, and enables alternative research projects to be developed for which the samples were not originally obtained. Sharing metagenomic datasets also allows significant scientific discoveries to be made (Dutilh et al., 2014), and restricting access to datasets can seriously delay scientific progress. For example, the Fourth Paradigm of data-driven scientific discovery describes how the advancement of a scientific field depends on how well researchers collaborate with one another (Hey et al., 2009).

However, there are several concerns when it comes to publishing diagnostic metagenomic datasets. For example, as we discuss here, misinterpretation can have serious consequences. Moreover, there is a possibility that pathogens could be missed by the original submitters, or even discovered at a later date.

Additionally, in human diagnostic settings sharing of metagenomic data becomes ethically complex due to issues of patient confidentiality and privacy infringements. For example, if human samples were obtained for a specific purpose like virus discovery, then ethical permissions may only be granted for that purpose alone. If a researcher were to investigate any other aspect of the metagenomic data – such as looking for a correlation between a human genetic mutation and infection status or outcome – this would be in breach of the original ethical approval and/or statutory obligations.

## Expectations of the End-User

Ultimately, a decision must be made on how to act upon the result of a diagnostic test. Such decisions are made every day by clinicians, veterinarians, ecologists, epidemiologists, scientists, farmers, and private citizens. Commercially available tests for pathogen detection often condense a reported result into a binary “presence or absence” call, with much of the technical detail being deliberately and carefully hidden, to make interpretation easy. Metagenomic analyses currently provide end-users with a “shotgun” picture of the microbiome that includes a list of the organisms that are theoretically present based on sequence similarity. Interpretation relies on an expert examining the data and making assessments based on their experience, particularly in regard to the reliability of the methodology, nuances between viral or bacterial strains, genetic similarities between viruses, bacteria, and eukaryotes, and the potential for contamination of genome databases (Gonzalez et al., 2014; Merchant et al., 2014) and nucleic acid extraction kits (Salter et al., 2014). Therefore, the reports currently generated for metagenomic datasets are not yet conducive to widespread use, as witnessed by the recent mass public reporting of the alleged detection of anthrax and the bubonic plague on the New York City subway system, based on a metagenomic analysis which contained potential but non-definitive hits to the these pathogens (Afshinnekoo et al., 2015; Mason, 2015; Yong, 2015).

At least in the US, no tests for human genetic testing are allowed to enter the market as medical devices without strict analytical and clinical validation to ensure consistent and robust results. One recent example is the case of the direct-to-consumer (DTC) human genotyping service provided by the company 23andMe. In November 2013, the 23andMe Personal Genome Service product was banned from marketing and providing “medical reports” of “health risks” and “drug response” when the company failed to deliver adequate evidence to the FDA for validation of specificity and sensitivity (Woods, 2013). Similarly, we see a big challenge for the metagenomics community to develop robust analysis methods before metagenomics can be approved for widespread clinical use. We note that, as of February 2015, the FDA has eased access to DTC DNA screening for several inherited diseases.

Another recent case illustrates the dangers of misinterpretation of metagenomic analysis (based on unpublished data). In this case, an automated metagenomic pipeline, MG-RAST (Meyer et al., 2008) was used to analyze a metagenomic dataset from environmental sample source. Prior to the data analysis, a worker involved in sampling was suffering from an undiagnosed illness. During the preliminary analysis a sequence hit to a Risk Group 3 pathogen was observed in the results from MG-RAST, and the group involved in the sampling suspected that this could be the cause of the worker’s illness. When the affected worker requested testing and prophylactic treatment for this notifiable zoonotic disease at a clinic, government organizations in human and animal health became involved. However, upon detailed review of the data, it was revealed that the “sequence hit” in MG-RAST actually matched a known archaeal contaminant in the draft genome assembly of the pathogen, rather than the pathogen itself. Although the contaminant sequences were clearly unrelated to the pathogen and had already been retracted from Genbank, they were still present in the automated annotation pipeline, which relied on an outdated database. We anticipate that such situations are likely to become increasingly common as metagenomics becomes mainstream.

It is our experience that when collaborators are presented with the taxonomic report from a metagenomic study for the first time, they may be overwhelmed. After some time spent studying the data, some even become skeptical. This is due to three factors: (i) the sheer enormity of microbial taxonomic diversity in any given sample, (ii) the confounding effect of gene conservation between taxonomic groups, and (iii) the potential for relative scarcity of reads representing a pathogen, even in samples with a significant viral load. Another example in our experience was revealed when a collaborator asked why a small number of cetacean sequence reads (derived from whales and dolphins) were present within the metagenomic data from an animal slaughterhouse that was processing cattle and sheep (Hall et al., 2013). This particular study was aimed at virus discovery, and was designed to be very sensitive, using sequence similarity search parameters that allowed identification of distantly related hits to enable the detection of novel viruses with low-level homology to known sequences. However, especially in eukaryotes, genetic sequences can be highly conserved, and in this case, the homologous matches occurred due to genetic conservation between the spuriously observed cetaceans, and ruminants such as the cattle and sheep processed in the slaughterhouse.

Explaining such cases to collaborating researchers takes time and careful communication, to allay their concerns about the inherent inaccuracies of the metagenomic method. Imagine, then, how difficult it could be to allay the concerns of a patient reading a report containing a non-significant hit to smallpox, or a farmer reading a report containing a spurious hit to foot-and-mouth disease.

## Considerations for Clinical Use of Metagenomics

Before incorporating metagenomics into routine clinical diagnostics, viral databases need to be vastly expanded, so that sequences can be more accurately annotated (Dutilh, 2014). Thus, efforts to map the complete viromes of humans and economically relevant animal or crop species will provide a baseline for allowing metagenomics to be applied in the clinic. Moreover, a good reference database allows novel viruses to be readily detected. Novel viruses that are observed in humans for the first time, e.g., after genomic recombination of known viruses or by zoonotic transfer from risk species like bats, can be flagged as potentially dangerous (Temmam et al., 2014).

Moreover, general considerations need to be made, such as the time taken to generate results, how these results are reported, how performance attributes like sensitivity are assessed, and how quality assurance programs and criteria for accreditation should be developed. Indeed, recent concerns about the potential for false-positive detection of pathogens in metagenomics datasets (Naccache et al., 2014; Rosseel et al., 2014) underscore the need to develop proper quality control procedures before routine deployment of metagenomics in the clinic or diagnostic laboratory.

Large-scale deployment of diagnostic methods in clinical laboratories is facilitated by simplicity, repeatability, low costs, established quality assurance programs, and quick turn-around times for the production of results. The time and cost of processing a sample through a next-generation sequencer, albeit rapidly reducing, is still prohibitive when considering large scale diagnostic testing. Additionally, metagenomics is currently too complex for immediate release into the diagnostic laboratory. There is a lack of standardization in the laboratory methods applied, such as the choice of sequencing platform or upstream sample preparation that is used. The *ad hoc* and heterogeneous tools currently employed for the analysis of high-throughput metagenomic datasets will also need to be further streamlined and unified. Reliability values are needed to account for the conservation of identifying sequences between pathogens and non-pathogens. In addition, depending on the methodology used, even a virus present in high titer might be represented by only a few reads in a metagenomic dataset. Thus, protocols need to be optimized, and in cases where coverage of a potential pathogen is low, other diagnostic methods, such as PCR, culture, immunoassays, or electron microscopy may be necessary to confirm the presence of the pathogen.

Nucleic acid amplification technologies (NAAT) such as real-time (quantitative) PCR already offer a rapid, cheap, sensitive and specific test for application in a broad range of settings (Gray and Coupland, 2014). Existing NAAT diagnostic tests already have a high degree of utility and meet diagnostic requirements for many areas – namely those requiring the detection of specific pathogens. Ultimately, the most valuable application of metagenomics in the clinic may be to replace serial testing/multiple single-plex assays, by offering a universal metagenomics-based test. Costs and turn-around times will still need to reduce significantly, and as mentioned above, quality assurance and method standardization are areas that will need major development. The Critical Assessment of Metagenome Interpretation^3^ (CAMI) is currently addressing these bioinformatic challenges in the form of a competition, by inviting tool developers to compete in the analysis of defined metagenomic datasets. CAMI evaluates the metagenome analysis tools and methods independently, comprehensively, and without bias. Thus, CAMI is paving the way for consistent and reliable metagenome interpretation tools to surface and receive international recognition.

## Etiology

Many end users will view metagenomics as a new technology, and in terms of application outside of a research setting, it certainly is. With the advent of new technologies come high expectations. For example, given the widely held apocryphal notion that many unsolved diseases are caused by viruses (Lipkin, 2014), will such diseases of unknown etiology now be resolved? Depending on the situation, this may or may not be the case, but it is certain that there are many other possible reasons for an unresolved etiology, such as an inadequate specimen or a diagnostic test that did not include the relevant pathogen, or a toxigenic or genetic cause of the disease.

One criticism of metagenomic virus discovery projects is that the mere detection of a micro-organism in a disease-state is not sufficient to establish etiology (Canuti et al., 2014). However, this holds equally true for any other detection protocol. There are certainly high profile instances of a “virus in search of a disease,” and of a “disease in search of a virus”. This has become particularly apparent for syndromic diseases such as encephalitis, where orthodox testing regimes have failed to identify a cause, and for cancers or autoimmune disease of unknown etiology. Evidence for the involvement of viruses in some cases of type 1 diabetes, inflammatory bowel disease, and asthma has recently been summarized (Foxman and Iwasaki, 2011). However, it is now generally recognized and accepted that viral metagenomics is primarily suitable as a discovery and detection method, and that any claims made in regard to etiology require extensive supporting information to fulfill Koch’s postulates, or variations thereof where applicable (Mokili et al., 2012).

Another criticism is that virus discovery, including viral metagenomics, is a descriptive research field that lacks hypotheses or the pursuit of knowledge about higher level biological processes. While this is a fair criticism, it should not be used to hinder or halt efforts to discover new viruses. The processes of infection, pathogenesis, or disease ecology cannot be fully understood without a basic fundamental description of the viral ecosystem, e.g., of the human virome in the case of the human body. Similarly, the development of therapeutics, vaccines, and culture methods may be informed by the discovery of new viruses. Highly divergent viral genomes may provide information about critically conserved genes and thus reveal targets for antiviral therapies, or epitopes for vaccine development. Finally, the notoriously prevalent “unknowns” in viral metagenomes can only be resolved if we face the grand challenge of mapping viral sequence space first (Dutilh, 2014).

## Conclusion

Virus discovery by metagenomics is still a fresh and developing field. Huge gains have already been made in the discovery of new viral species in a wide range of host species and samples (several examples can be found in the Frontiers in Virology Research Topic “Virus discovery by metagenomics: the (im)possibilities” of which this article is part). However, the application of viral metagenomics outside of the research setting remains relatively unexplored. Importantly, the issues surrounding the use of these methods to complement or replace existing clinical diagnostic tools need to be discussed in detail. The increase in sequencing efforts to characterize the viromes of various host species will lay a foundation for further analysis of viral metagenomes by providing a reliable reference database. To facilitate this, metagenomic datasets need to be made publicly available and mined (Dutilh, 2014), but at the same time this needs to be balanced against ethical, legal, and cultural factors and potentially include filtering steps to remove sequences matching the human reference, as a safeguard for the privacy of the individual. Incidental detection or spurious reporting of viruses, especially those of high consequence for health or trade, will require special consideration. Moreover, any application of viral metagenomics outside of a research project, for example for use in the clinic, will require good communication between patients, clinicians, and scientists, including informed consent about the handling and reporting of any incidental findings. In the case of agriculture, similar communication and agreement is required between the production sector, veterinarians, and regulators. We present a summary of the seven main points of concern and our proposed actions for resolving these in **Table 1**.

**Table 1:** A summary of seven major issues identified when considering the use of metagenomics as a diagnostic method, and the proposed actions that could resolve these issues.

Issue	Description of problems	Proposed actions to resolve
(1) Handling of incidental findings	Incidental detection of a pathogen that is unrelated to the investigation is possible when using metagenomics. This may be of high consequence for a patient or industry. (For industry, see point 2 below.)	Adopt protocols used for incidental findings from medical imaging studies (magnetic resonance imaging, MRI) or genome sequencing. The clinician and patient should understand the potential for incidental findings, and a plan should be in place for acting on findings as required.
(2) Agricultural/Horticultural Implications for trade	Pathogens affecting industry or trade may be detected or suspected. Even unsubstantiated reports of a high risk pathogen can have deleterious economic effects.	Independent and accredited diagnostic methods should be used to confirm the finding. Regulatory authorities should be contacted early to raise these issues.
(3) Host genome	Host genome sequence may be present in clinical metagenomic datasets. This may contravene ethical approval or legislation for handling human genome sequence (depending on jurisdiction).	Bioinformatic filtering of the host genome or restricted data access may provide some protection. Ethics committees and repositories should develop guidelines for the handling of potentially personally identifiable data in the metagenomics data.
(4) Data sharing	Deposition of metagenomic datasets from clinical samples into public databases may be problematic due to conflict with ethical, privacy, and legal concerns.	Sharing of metagenomic data is critical to the advancement of scientific understanding. However, legal and ethical constraints need to be considered and appropriate measures taken, e.g., review by ethics boards and sharing through of an appropriate data-sharing repository.
(5) Cost	Next-generation sequencing is still costly when compared to conventional diagnostic testing, especially for detecting known pathogens.	We expect that sequencing costs will continue to drop. Metagenomics is already cheaper than performing a large series of specific tests, but conventional diagnostic methods may still be preferred when searching for specific targets.
(6) Quality assurance	Currently, there are no standardized metagenomic methods: sample processing, sequencing instruments, bioinformatic analyses, and reporting of results all vary widely.	Guiding authorities will need to consider the role of metagenomics in diagnostic testing and provide protocols and quality assurance programs. For bioinformatic interpretation, the Critical Assessment of Metagenome Interpretation (CAMI) paves the way by evaluating methods.
(7) Etiology	The detection of a micro-organism in a sample does not necessarily mean it has caused the disease.	As with all diagnostic assays, prior evidence of pathogenicity or further study to determine causation (Lipkin, 2010) will be necessary to conclude that a specific organism is causing the disease.

History shows that care is required when delivering a new and potentially disruptive technology. No doubt, larger debate and more deeply held concerns lie ahead in the area of sequencing eukaryotic genomics, especially for the human genome, but it is worth beginning the discussion on where viral metagenomics is heading, beyond research, on the way towards application of viral metagenomics in the field and clinic.

## Conflict of Interest Statement

The authors declare that the research was conducted in the absence of any commercial or financial relationships that could be construed as a potential conflict of interest.

## Footnotes

1 http://www.pathogenica.com/

2 http://www.aperiomics.com/

3 http://www.cami-challenge.org/
